# Chesapeake DolphinWatch sightings data (2017–2022): Citizen science reports of bottlenose dolphins observed in the Chesapeake Bay, USA

**DOI:** 10.1016/j.dib.2024.110368

**Published:** 2024-03-23

**Authors:** Lauren K. Rodriguez, Jamie C. Testa, Kirsten Silva, Helen Bailey

**Affiliations:** a Chesapeake Biological Laboratory, University of Maryland Center for Environmental Science, Solomons, MD 20688. USA; b Applied Animal Ecology Research Unit, Department of Zoology, University of Innsbruck, 6020 Innsbruck, Austria; c Blue Wave Consulting LLC, Great Mills, MD 19410. USA

**Keywords:** Tursiops truncatus, Marine mammals, Estuary, Volunteer science, Monitoring

## Abstract

Atlantic bottlenose dolphins are extensively studied, though little has been published regarding their occurrence patterns in the large and highly urbanized estuary of the Chesapeake Bay, USA. To address this knowledge gap, the Chesapeake DolphinWatch project was initiated in the summer of 2017. Utilizing a citizen science (also known as volunteer science) methodology, members of the public were encouraged to report dolphin sightings through a specialized mobile (iOS and Android) and web-based (https://chesapeakedolphinwatch.org) application. This approach ensured extensive, yet non-invasive and financially-efficient, data collection. The dataset presented here includes bottlenose dolphin sighting reports submitted to Chesapeake DolphinWatch by citizen scientists over five years; from June 28, 2017 through December 9, 2022. These data have been quality checked by researchers at the University of Maryland Center for Environmental Science's (UMCES) Chesapeake Biological Laboratory (CBL) in Solomons, Maryland (USA). This dataset holds potential for various applications, such as analyzing the spatiotemporal patterns of dolphin presence within the Chesapeake Bay, investigating the behavior and movements of bottlenose dolphins in the mid-Atlantic, and serving as a comparative benchmark for studies in other estuarine systems. By integrating community engagement with technological platforms, the provided data showcases the invaluable role of citizen science in advancing marine ecological research.

Specifications Table

**Table:** 

Subject	Biological Sciences
Specific subject area	Marine Biology, Ecology and Behavior
Data format	Raw
Type of data	Table
Data collection	Collection and confirmation of reported sightings of bottlenose dolphins began in June 2017 when the Chesapeake DolphinWatch web-based application (https://chesapeakedolphinwatch.org/) was launched to the public. The mobile application was released in May 2018 in which sightings were then reported from both interfaces. Users of both platforms must register for an account to submit a sighting report. Sighting reports include information from the user account as well as details from the sighting: date, time, location, approximate number of dolphins, a brief description, and/or photos/videos.
Data source location	Dolphin sighting reports were able to be reported internationally, however, most were in the Chesapeake Bay, the region in which the application was developed.
Data accessibility	Repository name: Mendeley Data identification number: doi: 10.17632/ntvpz3rhn4.1 Direct URL to data: https://data.mendeley.com/datasets/ntvpz3rhn4/1
Related research article	[1] L.K. Rodriguez, A.D. Fandel, B.R. Colbert, J.C. Testa, H. Bailey, Spatial and temporal variation in the occurrence of bottlenose dolphins in the Chesapeake Bay, USA, using citizen science sighting data. PLoS ONE (2021) 16(5): e0251637. https://doi.org/10.1371/journal.pone.0251637

## 1 Value of the Data

• **Knowledge:** This is the first published dataset regarding the precise spatial presence of bottlenose dolphins throughout the Chesapeake Bay estuary. This data provides spatiotemporal information from June 2017 through June 2022 and is freely available to all interested users.
• **Conservation:** Dolphins in the western North Atlantic are considered depleted following the unusual mortality event (UME) that occurred in 2013–2015 [2]. In the Chesapeake Bay, bottlenose dolphins face challenges from high densities of recreational and commercial vessels, naval operations, and relatively low water quality. Historically, despite being protected under the US Marine Mammal Protection Act of 1972 [3], bottlenose dolphins were not recognized as seasonal residents of the Bay. This oversight impacts species management efforts such as stock assessments, monitoring by regulatory agencies, and implementation of measures for reducing incidental fisheries bycatch. Our data collection efforts aim to rectify this gap, enhancing our understanding of the distribution of bottlenose dolphins in this highly-urbanized region and informing targeted management strategies.
• **Management:** A subset of data from Chesapeake DolphinWatch have been used in an open-access, peer-reviewed manuscript which modeled the spatial and temporal presence of bottlenose dolphins throughout the Chesapeake Bay [1,4]. This manuscript was referenced in an Environmental Impact Statement report generated by the US Naval Air Station in the Patuxent River Complex (Maryland, USA; www.PRCEIS.com). In previous Environmental Impact Statement (EIS) reports, the US Naval Air Station in the Patuxent River Complex had only acknowledged harbour seals as a marine mammal in middle Chesapeake Bay. Due to dolphin sighting reports from Chesapeake DolphinWatch submitted during the public comment period, they are now considering bottlenose dolphins as a marine mammal which frequents the Chesapeake Bay, inherently increasing their awareness of the seasonal presence of this protected species. The latest EIS now includes the seasonality of bottlenose dolphins in both lower and middle Chesapeake Bay.
• **Public engagement:** Citizen scientists who contribute to Chesapeake DolphinWatch are encouraged to engage with scientific research projects occurring in their region, furthering environmental stewardship throughout states which border the Chesapeake Bay's 11,684-mile shoreline in Maryland, Virginia, Delaware, and Washington, D.C.
• **Future research:** This data set provides a foundation for future and expanded research regarding future species distribution or ecosystem models in our study region.

## 2 Background

**Fig. 1:**
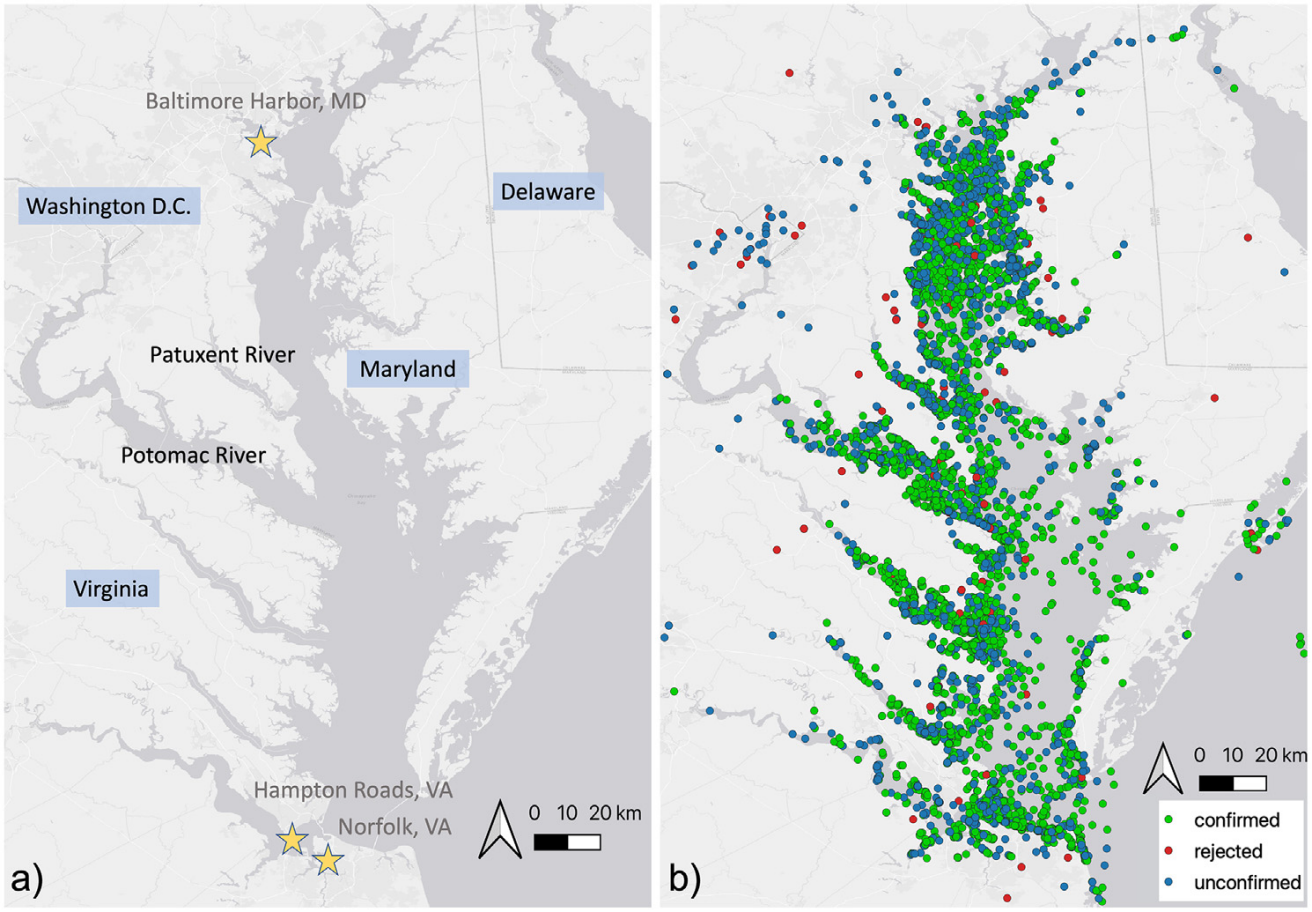
a) Map of the Chesapeake Bay. States (highlighted in blue) and major tributaries are labeled. Stars indicate the locations of large shipping ports and the largest naval base in the world (Norfolk, VA). b) Chesapeake DolphinWatch user sighting reports 2017–2022 represented by points colored by their confirmation status.

Chesapeake DolphinWatch is a citizen science program which monitors bottlenose dolphin (*Tursiops truncatus*) presence throughout the Chesapeake Bay (Fig. 1a). Beginning in 2017, the main objective of Chesapeake DolphinWatch (also referred to as ‘DolphinWatch’) is twofold: firstly, to engage a network of citizen science volunteers to actively participate in scientific observation and secondly, to utilize the observations from this network to aid researchers in identifying the seasonal, intrannual, and geographic patterns of bottlenose dolphin occurrence in and around the Chesapeake Bay (Fig. 1b). Since its inception, the program has garnered significant attention, amassing over 14,000 registered users and receiving more than 7,000 dolphin sighting reports.

The University of Maryland Center for Environmental Science (UMCES) issued a press release when the app was launched in 2017. This press release garnered attention from local and regional news outlets to encourage DolphinWatch user registration. Attention from news outlets continued garnering public attention after the initial press release. Press releases were also issued annually and UMCES staff conducted multiple media interviews every year to raise awareness about the project and recruit volunteers. DolphinWatch staff also created social media accounts to recruit citizen scientists and app users as well as share information on cetaceans, research insights, and project updates. DolphinWatch staff were often asked to attend and speak at outreach events in the local and regional community. This program is also featured at the Chesapeake Biological Visitor Center in Solomons, Maryland, USA.

Volunteers were not financially rewarded for their contributions to the data. DolphinWatch staff have provided information to the volunteers via annual summaries on the UMCES website (https://www.umces.edu/dolphinwatch), published journal articles with open access, and sharing of images and information on social media.

In 2021, a portion of this data (spanning 2017–2019) was utilized to analyze dolphin occurrences in the Bay, as detailed in a published article [1]. However, that analysis broadly aggregated sightings into regions (Lower, Middle, and Upper Chesapeake Bay; [4]). The dataset presented here, in contrast, offers detailed geographic coordinates (latitude/longitude) for each sighting. This granular data provides a more precise spatial representation of dolphin presence, enhancing the understanding of their distribution patterns within the Bay.

## 3 Data Description

All herein descriptions of the data correspond to information that are in the data file “Chesapeake_DolphinWatch_Data_2017to2022.csv”.

**Fig. 2:**
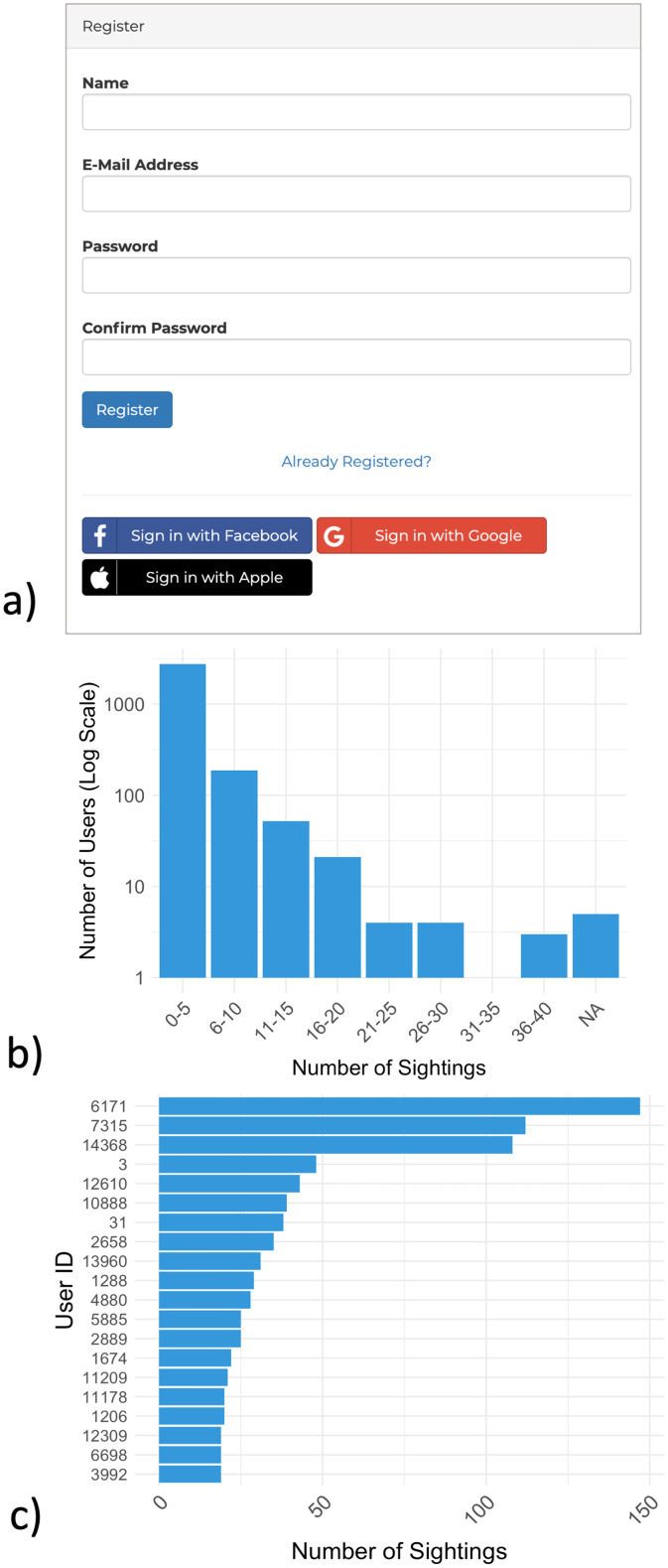
a) Chesapeake DolphinWatch user registration form. b) Distribution of confirmed sightings submitted per registered user. c) Top twenty registered users based on the number of confirmed sightings that they have submitted to Chesapeake DolphinWatch.

Upon account registration, DolphinWatch users were required to register with their email address and a name in the event they needed to be contacted for additional information regarding their sighting(s) as part of the data validation process. Users can also sign in with their Facebook, Google, or Apple account (Fig. 2a). For the purpose of anonymity, users were assigned a unique number, which is listed in the “User_no” variable of the attached dataset. Most users submitted 0–5 confirmed sightings during their registration period from 2017 to 2022, however, a small subset of users have submitted over 30 sightings (Fig. 2b & c).

**Fig. 3:**
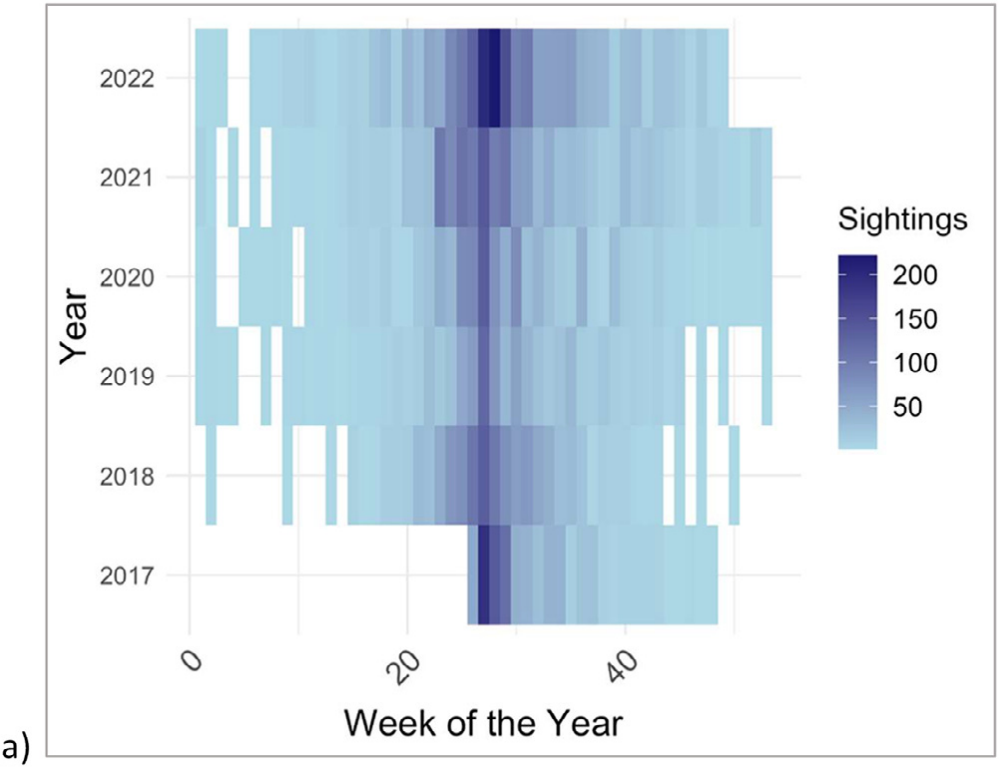
Heatmap showing the frequency of weekly sightings per year.

Though DolphinWatch was launched in 2017 via a web-based platform only, this dataset includes the culmination of sightings submitted to both the web-based and mobile app (which was launched in May 2018). The “Date” variable in this dataset is formatted: Month/Day/Year, which was automatically formatted when users submitted a sighting report. Most sighting reports were submitted during the summer season (weeks 25–35) each year (Fig. 3). July 4, a federal holiday and thus a very popular day for boaters to be on the water, falls during the 27th week of the year and was usually the most popular day of dolphin sighting report submission.

All sighting reports submitted to Chesapeake DolphinWatch require the location of the sighting as coordinate points (latitude and longitude). This coordinate location could either be acquired directly via the mobile device (e.g. the user's current location) or could be selected on a dynamic map by the application user. Most sightings reported were in the mainstem of the Chesapeake Bay or its tributaries (Fig. 1b) though users occasionally entered sightings outside of this area as the app does not restrict sighting reports based on the location in which they are reporting.

**Fig. 4:**
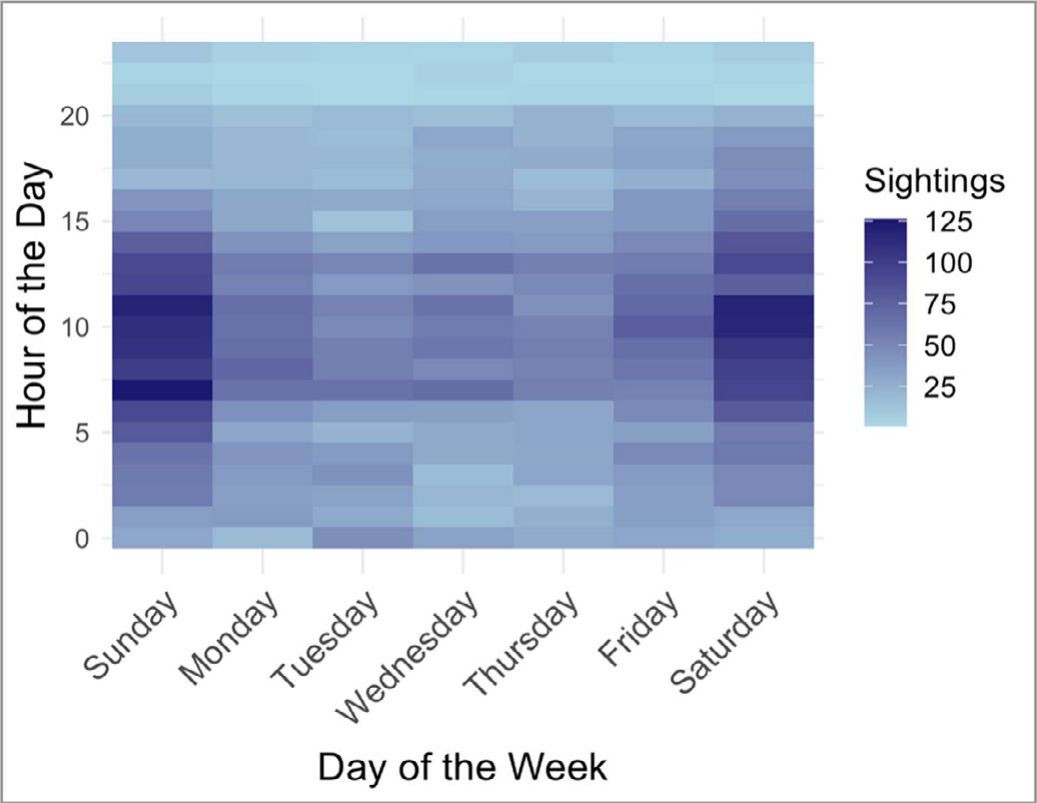
Heatmap of confirmed sighting report activity (on average) during the week.

The “Time” (Hour:Minutes AM/PM) of each sighting was also uploaded via the mobile device and could be edited by the user to reflect the actual time of dolphin sighting if reported retrospectively. In the dataset provided, the “Time” variable was transformed to be on a 0–23 hour scale, negating the requirement for AM/PM labeling. Most confirmed sighting reports were submitted during the hours of 6–15, especially on weekends (Fig. 4).

The application user was prompted to submit an estimate of group size using a sliding bar scale with numerical bins (‘fewer than 5′, ‘5–10′, ‘10–50′, ‘50–100′, ‘more than 100′). In 2017, a maximum group size option for users to select was ‘more than 10′, but was removed in 2018 from the application after larger groups were regularly reported. Therefore, data from 2017 may have ‘more than 10 (legacy data)’ listed for the “Size” variable.

Furthermore, sighting reports from 2018 included a section where additional text details (i.e. behaviors, movements, environmental conditions) and media (pictures/videos) could be uploaded. Text details and media are not included in this dataset for the purpose of standardization and user data privacy.

**Fig. 5:**
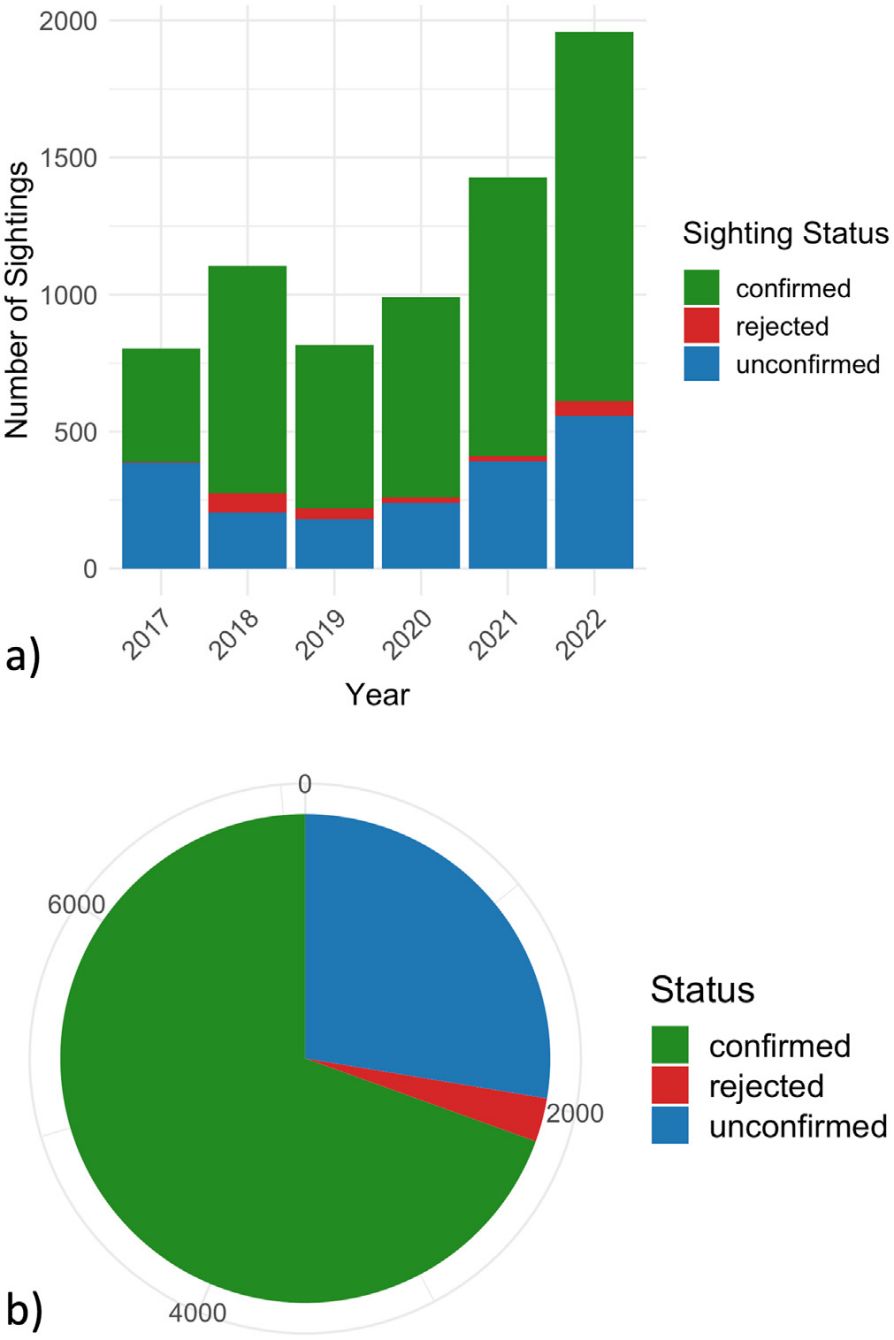
a) Confirmation status of sighting reports submitted each year. b) Confirmation status of sighting reports described in this dataset (total sightings *n* = 7693).

The “Status” of each report indicates whether the dolphin sighting had been ‘confirmed’, ‘rejected’, or ‘unconfirmed’ by members of the Chesapeake DolphinWatch scientific team. The status variable is automatically set to ‘unconfirmed’ by the application upon submission of the user's sighting report. Some sightings remain unconfirmed by the team due to lack of information (i.e. lack of descriptive comments or media of the dolphin sighting event). Users who report ‘unconfirmed’ sightings then receive an automated email after one month asking them to provide a description or image of their dolphin encounter. On average, most sightings were confirmed with approximately 25% remaining unconfirmed (Fig. 5a & b).

## 4 Experimental Design, Materials and Methods

Visual sightings of dolphins were submitted by members of the Chesapeake Bay community (residents and visitors to Maryland, Washington D.C., and Virginia) in the Chesapeake DolphinWatch application (ChesapeakeDolphinWatch.org). Volunteer community members were not in contact with the Chesapeake DolphinWatch team prior to or during their sighting events. They did not undergo any training by UMCES scientists prior to their sighting events.

**Fig. 6:**
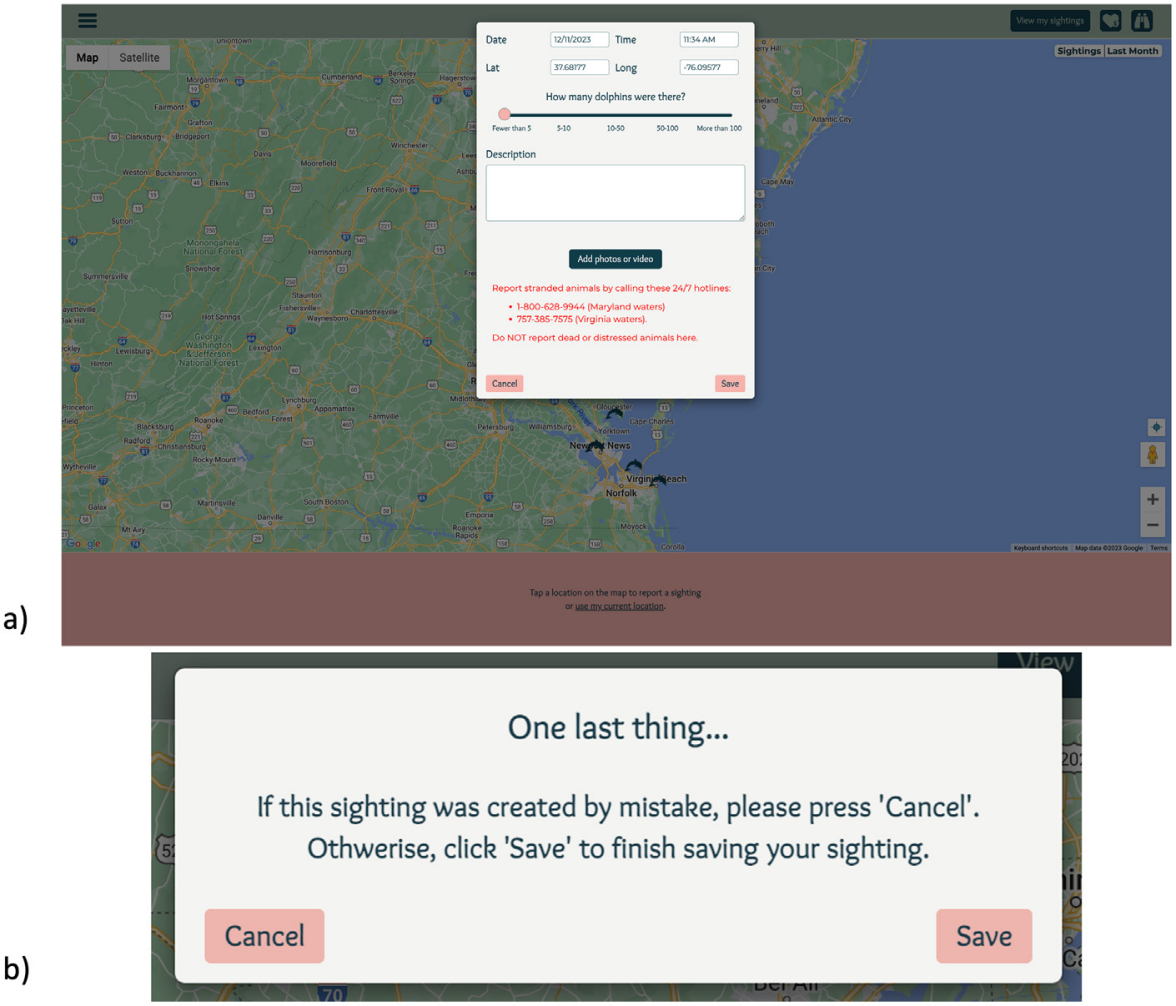
a) Upon entering their sighting report information, b) users were prompted with a notification ensuring that their sighting report is accurate.

Once logged into the app, users were brought to an interactive map and clicked on the map in the location where dolphins were sighted which opened a sighting report form in a popup box (Fig. 6a). Once they completed the sighting report, users clicked the “Save” button. Before the submission of their sighting, users were prompted to ensure that their sighting report was fully accurate and clicked save (Fig. 6b).

Participating members of Chesapeake DolphinWatch were voluntary observers who saw dolphins opportunistically and were not specifically permitted to do scientific research. Upon logging into the application, DolphinWatch users were shown tips for safe wildlife viewing as well as information on who to contact if a dead or stranded animal was sighted. Those data on stranded animals were ‘rejected’ by DolphinWatch staff during the data validation process and were not included in the database.

All data go through our standard dolphin sighting verification procedure (see Supplemental Information 2 from [1]) which is carried out by trained staff at the University of Maryland Center for Environmental Science Chesapeake Biological Laboratory.

Participating members of Chesapeake DolphinWatch were also instructed to comply with NOAA Marine Life Viewing Guidelines (https://www.fisheries.noaa.gov/topic/marine-life-viewing-guidelines) to prevent the disturbance of marine species protected by the Marine Mammal Protection Act. These guidelines were automatically displayed to users upon logging into the Chesapeake DolphinWatch application.

## Limitations

### User-related

**Fig. 7:**
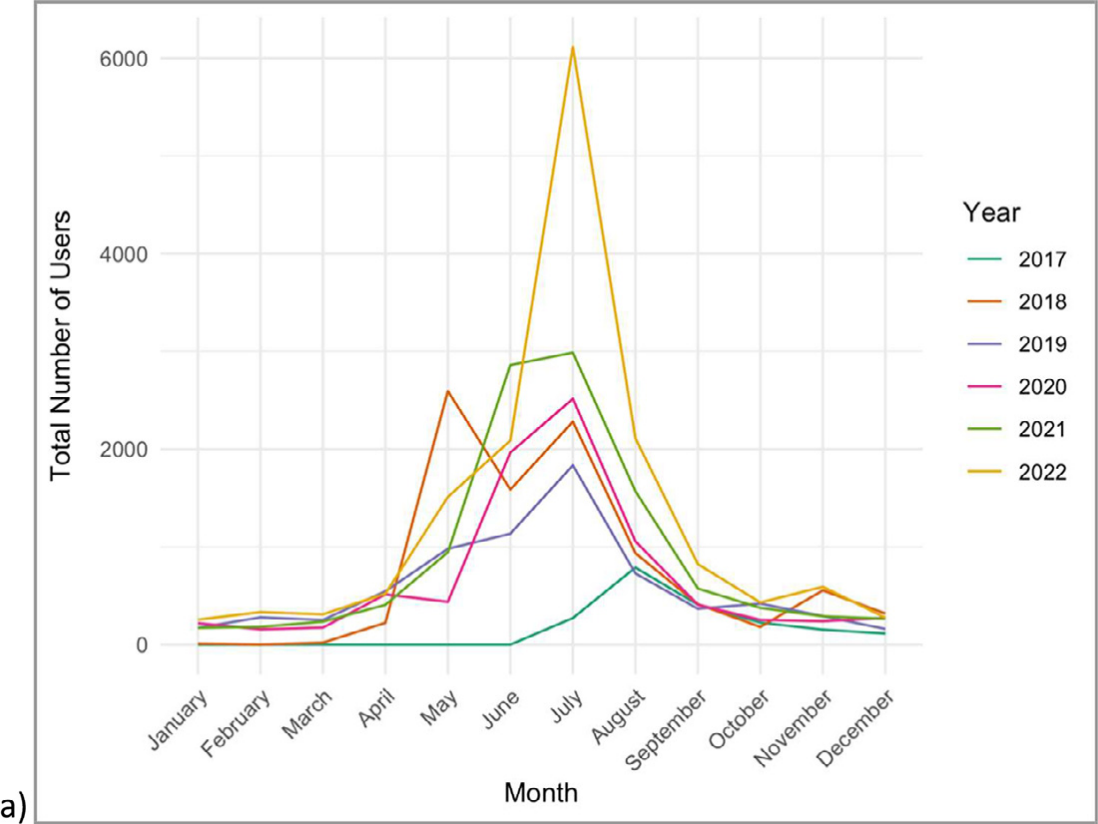
Google Analytics data showing the frequency of Chesapeake DolphinWatch users per year (2017–2022).

Location and temporal data may not reflect the actual sighting date/time/place due to miscellaneous user and/or device errors. Sighting reports also may reflect the distribution of citizen scientist reporters rather than absolute dolphin presence, as indicated by Google Analytics data regarding website and mobile application traffic (Fig. 7& Table 1). Therefore, an absence of sighting reports does not necessarily indicate the absence of dolphins.

**Table 1:** Summary statistics of Google Analytics data from 2017 to 2022.

Year	Application Platform	Average Session Duration (MM:SS)	Returning Visitors/Users (%)	New Visitors/Users (%)
2017	Web	02:21	21.7	78.3
	Mobile	NA	NA	NA
2018	Web	02:43	22.1	77.9
	Mobile	03:07	44.7	55.3
2019	Web	02:11	21.5	78.5
	Mobile	02:11	38.8	61.2
2020	Web	02:07	20	80
	Mobile	02:07	37.5	62.5
2021	Web	01:48	19.7	80.3
	Mobile	01:48	34.5	65.5
2022	Web	01:26	16.6	83.4
	Mobile	01:26	30	70

### Technical

The web version of Chesapeake DolphinWatch was offline for maintenance and upgrades from December 15, 2017 through April 1, 2018 while a freely available mobile application for Android and iOS devices was developed.

Occasionally an error caused the app to crash, therefore, short term unavailability of the DolphinWatch application went unreported from 2017 to 2022, which may have impacted the ability of users to submit their dolphin sightings. Known application failures are listed in Table 2.

**Table 2:** Application errors and outages from 2017- 2022.

Date/time app reported down	Date/time app was restored	Estimated time app was unavailable	How outage was discovered	Notes from software developer (i.e.-cause, solution)
8/22/2018	8/22/2018	Uncertain - possibly 2 days	Staff tried to log onto back end to validate sightings and received a server error.	User manually entered a latitude and longitude which had more decimal places than the database supports. This caused an issue which broke the query that gathered all of the sightings for both admin and the map page. To resolve this, the sighting in question was deleted and reuploaded with the latitude and longitude input rounded to the 7th decimal place. Higher precision than that should not be needed since it would be nearly impossible to distinguish on Google Maps
7/12/2020	7/13/2020	1 day	Staff tried to log onto the back end to validate sightings and received a server error.	User reported a sighting with nothing in the location field for lat/long. It caused the front and back ends to crash. Software developers set a feature to prevent another app crash in the future.
7/17/20 3:59PM	7/20/2020 4:46PM	3 days	Emails from users on front end and staff working in map on back end	N/A
8/20/20 2:33PM	8/20/20 at 6:00pm	<1 day	Staff tried to log onto the back end to validate sightings and received a server error.	Someone entered a sighting when the on-water API (which says if the sighting is on land or in the water) was having an outage which caused the app to crash. Software developers implemented a control to prevent app crash in the event of an on-water API outage.
7/2/2021 3:02PM	7/5/2021 3:17PM	< 1 day	Staff tried to log onto the back end to validate sightings and received a server error.	N/A
8/12/21 4:48PM	8/12/2021 4:55PM	< 1 day	Staff tried to log onto the back end to validate sightings and received a server error.	N/A

## Ethics Statement

Informed consent was obtained from application users during their Chesapeake DolphinWatch account registration. Usernames as well as email addresses have been anonymized in the final dataset file. This research did not involve any human or animal experiments.

## CRediT authorship contribution statement

**Lauren K. Rodriguez:** Conceptualization, Formal analysis, Writing – original draft, Visualization. **Jamie C. Testa:** Software, Validation, Data curation, Writing – review & editing, Project administration. **Kirsten Silva:** Validation, Data curation, Writing – review & editing. **Helen Bailey:** Conceptualization, Software, Writing – review & editing, Supervision, Project administration, Funding acquisition.

## Data Availability

Chesapeake DolphinWatch sightings data (2017-2022): Citizen science reports of bottlenose dolphins observed in the Chesapeake Bay, USA (Original data) (Mendeley Data).
